# Comparison of Antioxidant Effects of Honey, Glibenclamide, Metformin, and Their Combinations in the Kidneys of Streptozotocin-Induced Diabetic Rats

**DOI:** 10.3390/ijms12010829

**Published:** 2011-01-21

**Authors:** Omotayo Owomofoyon Erejuwa, Siti Amrah Sulaiman, Mohd Suhaimi Ab Wahab, Sirajudeen Kuttulebbai Nainamohammed Salam, Md Salzihan Md Salleh, Sunil Gurtu

**Affiliations:** 1 Department of Pharmacology, School of Medical Sciences, Universiti Sains Malaysia, 16150 Kubang Kerian, Kelantan, Malaysia; E-Mails: sbsamrah@kb.usm.my (S.A.S.); msuhaimi@kb.usm.my (M.S.A.W.); 2 Department of Chemical Pathology, School of Medical Sciences, Universiti Sains Malaysia, 16150 Kubang Kerian, Kelantan, Malaysia; E-Mail: sirajuden@kb.usm.my; 3 Department of Pathology, School of Medical Sciences, Universiti Sains Malaysia, 16150 Kubang Kerian, Kelantan, Malaysia; E-Mail: matledpb@yahoo.com; 4 School of Medicine and Health Sciences, Monash University Sunway Campus, Jalan Lagoon Selatan, 46150, Bandar Sunway, Selangor, Malaysia; E-Mail: sgurtu@gmail.com

**Keywords:** diabetes mellitus, streptozotocin, oxidative stress, antioxidant enzymes, kidney, tualang honey, metformin, glibenclamide

## Abstract

Hyperglycemia-induced increase in oxidative stress is implicated in diabetic complications. This study investigated the effect of metformin and/or glibenclamide in combination with honey on antioxidant enzymes and oxidative stress markers in the kidneys of streptozotocin (60 mg/kg; intraperitoneal)-induced diabetic rats. Diabetic rats were randomized into eight groups of five to seven rats and received distilled water (0.5 mL); honey (1.0 g/kg); metformin (100 mg/kg); metformin (100 mg/kg) and honey (1.0 g/kg); glibenclamide (0.6 mg/kg); glibenclamide (0.6 mg/kg) and honey (1.0 g/kg); metformin (100 mg/kg) and glibenclamide (0.6 mg/kg); or metformin (100 mg/kg), glibenclamide (0.6 mg/kg) and honey (1.0 g/kg) orally once daily for four weeks. Malondialdehyde (MDA) levels, glutathione peroxidase (GPx) and superoxide dismutase (SOD) activities were significantly elevated while catalase (CAT) activity, total antioxidant status (TAS), reduced glutathione (GSH), and GSH:oxidized glutathione (GSSG) ratio was significantly reduced in the diabetic kidneys. CAT, glutathione reductase (GR), TAS, and GSH remained significantly reduced in the diabetic rats treated with metformin and/or glibenclamide. In contrast, metformin or glibenclamide combined with honey significantly increased CAT, GR, TAS, and GSH. These results suggest that combination of honey with metformin or glibenclamide might offer additional antioxidant effect to these drugs. This might reduce oxidative stress-mediated damage in diabetic kidneys.

## 1. Introduction

Diabetes mellitus is one of the five main causes of death in the world [1]. The most common form of diabetes, which accounts for 90–95% of all diabetic cases, is type 2 diabetes [2]. It is a metabolic disorder characterized by hyperglycemia as a result of insulin resistance. This is often followed by β-cell dysfunction caused by inability of the pancreatic β-cells to compensate for the reduced insulin action [2]. The global prevalence of diabetes mellitus was estimated as 171 million people in 2000 and this figure is predicted to increase to 366 million by 2030 [3]. Besides its severe medical implications, this global projection has financial consequences due to the costs of managing this disorder and its associated complications. Since diabetes mellitus is a heterogeneous disorder with multiple causes, the beneficial effects of combined therapeutic agents aimed at specific patho-biological pathways of diabetes and its complications have been reported [4,5].

The pharmacological agents currently employed, such as sulfonylureas (e.g., glibenclamide), biguanides (e.g., metformin), thiazolidinediones (e.g., pioglitazone) and α-glycosidase inhibitors (e.g., acarbose) act to selectively modulate a specific pathological pathway [6,7]. As a result, these drugs control blood glucose levels provided they are regularly administered. Even though these drugs may be valuable in the management of diabetes mellitus, they have limitations due to undesirable adverse effects such as hypoglycemia, weight gain, secondary failure, and inability to arrest pancreas degeneration [8–10] or diabetic complications which have been linked to oxidative stress [11]. In view of the compelling evidence for a major role of oxidative stress in the development, progression, and complications of diabetes, antioxidants may serve as a potential therapy for ameliorating these [12,13]. Thus, an ideal therapy for diabetes mellitus would be a drug that not only possesses antihyperglycemic effect, but also enhances or protects the antioxidant defense system which is usually compromised. Unfortunately, among the currently available hypoglycemic agents, the choice is very limited.

In our previous studies, we have confirmed the hypoglycemic effect of Malaysian tualang honey as well as its antioxidant effect in the kidneys and pancreas of diabetic rats [14,15]. Based on literature, no study had investigated the effects of glibenclamide, metformin or both in combination with an antioxidant (honey) in the kidneys of experimental diabetic rats. This present study investigated and compared the role of honey as an adjunct to metformin and/or glibenclamide in relation to the activities of antioxidant enzymes and other markers of oxidative stress in the kidneys of streptozotocin (STZ)-induced diabetic rats.

## 2. Results and Discussion

Table 1 shows the effects of honey, metformin, glibenclamide, and their combinations on the fasting blood glucose and activities of antioxidant enzymes in the kidneys of control and streptozotocin-induced diabetic rats. The diabetic control rats (without drugs) had significantly elevated blood glucose levels compared to the non-diabetic rats. All the drugs significantly decreased the levels of blood glucose in diabetic rats. The non-diabetic rats supplemented with honey did not show significant changes in the activities of antioxidant enzymes, whereas a significant (*p* < 0.01) decrease of catalase (CAT) activity and increase of glutathione peroxidase (GPx) and superoxide dismutase (SOD) were observed in the STZ-treated rats. Treatment of diabetic rats with metformin, glibenclamide or their combination with honey significantly (*p* < 0.05) reduced SOD activity. Treatment of diabetic rats with metformin or glibenclamide did not produce any significant effects on CAT and glutathione reductase (GR) activities compared to diabetic control rats. In contrast, metformin or glibenclamide combined with honey showed significantly (*p* < 0.05) increased CAT and GR activities in diabetic rats compared to the diabetic control rats. No significant change in the activity of glutathione-S-transferase (GST) was observed in any group.

The results of the effects of honey, metformin, glibenclamide and their combinations on markers of oxidative stress in the kidneys of control and STZ-induced diabetic rats are shown in Table 2. The table reveals that total antioxidant status (TAS), reduced glutathione (GSH) and GSH/oxidized glutathione (GSSG) ratio were significantly (*p* < 0.05; *p* < 0.01) reduced while malondialdehyde (MDA) concentrations were significantly (*p* < 0.01) elevated in the STZ-treated rats (Table 2). Administration of honey, metformin, glibenclamide and their combinations to diabetic rats significantly (*p* < 0.05) restored the levels of MDA. Besides, all the agents as well as their combinations improved GSH/GSSG ratio although not significantly. Combination of metformin or glibenclamide with honey significantly (*p* < 0.05) increased TAS and GSH concentration.

**Figure 1:**
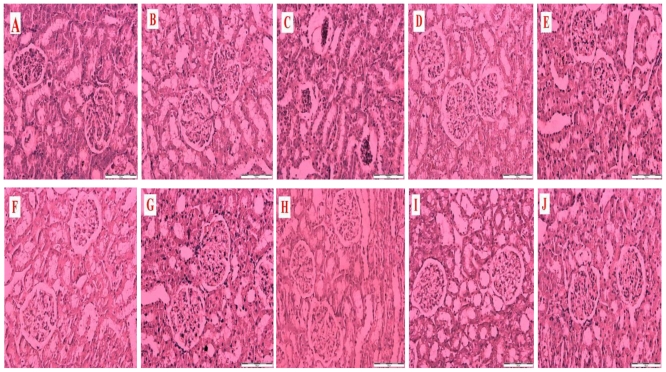
Representative photomicrographs of hematoxylin and eosin staining of the kidney (scale bar: 100 μm) of non-diabetic control rats treated with distilled water (**A**); non-diabetic rats treated with tualang honey (**B**); diabetic control rats treated with distilled water (**C**); diabetic rats treated with tualang honey (**D**); diabetic rats treated with metformin (**E**); diabetic rats treated with metformin and tualang honey (**F**); diabetic rats treated with glibenclamide (**G**); diabetic rats treated with glibenclamide and tualang honey (**H**); diabetic rats treated with metformin and glibenclamide (**I**); diabetic rats treated with metformin, glibenclamide and tualang honey (**J**).

Figure 1 is a representative photomicrograph of kidney sections showing the effects of honey, metformin, glibenclamide and their combinations on structural and morphological changes in kidneys of control and streptozotocin-induced diabetic rats. The section in Figure 1A is from a non-diabetic control kidney treated with distilled water showing normal morphological structures of the renal tubules, glomeruli and basement membrane. Figure 1B is a section of a non-diabetic kidney treated with tualang honey showing normal renal morphological structures similar to Figure 1A. Figure 1C is a section from a diabetic control kidney characterized by necrosis of the epithelium, thickness of the glomerular basement membrane and mesangial matrix expansion. Figure 1(D–J) reveals sections from diabetic kidneys treated with tualang honey (D), metformin (E), metformin and tualang honey (F), glibenclamide (G), glibenclamide and tualang honey (H), metformin and glibenclamide (I) or metformin, glibenclamide and tualang honey (J). Figure 1(D–J) showed less damage, improvement and restoration of renal cellular components compared to Figure 1C.

**Figure 2:**
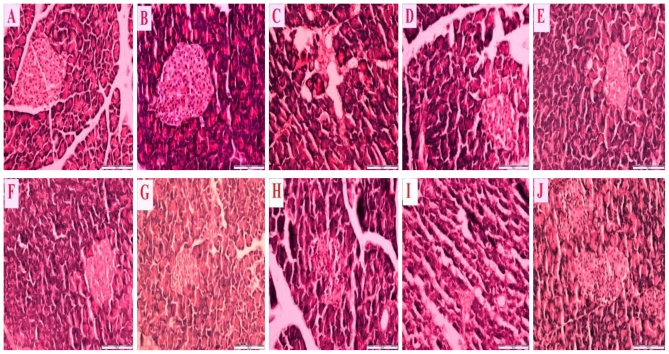
Representative photomicrographs of hematoxylin and eosin staining of pancreas (scale bar: 100 μm) showing non-diabetic control rats treated with distilled water (**A**); non-diabetic rats treated with tualang honey (**B**); diabetic control rats treated with distilled water (**C**); diabetic rats treated with tualang honey (**D**); diabetic rats treated with metformin (**E**); diabetic rats treated with metformin and tualang honey (**F**); diabetic rats treated with glibenclamide (**G**); diabetic rats treated with glibenclamide and tualang honey (**H**); diabetic rats treated with metformin and glibenclamide (**I**); diabetic rats treated with metformin, glibenclamide and tualang honey (**J**).

Figure 2 is a representative photomicrograph which shows sections of the pancreas of control and streptozotocin-induced diabetic rats treated with honey, metformin and/or glibenclamide as well as their combinations. Figure 2A reveals a section of normal pancreas treated with distilled water showing a normal cellular population in the islet of Langerhans. Figure 2B is a section of a normal pancreas treated with tualang honey, also showing normal architecture and cellular population, similar to in Figure 2A. Figure 2C is a section of a streptozotocin-induced diabetic control pancreas showing severe damage to the islet of Langerhans, pancreatic necrosis, reduced islet size, vacuolization and a reduced number of cells. Figure 2(D–J) reveals sections of streptozotocin-induced diabetic pancreas treated with tualang honey (D), metformin (E), metformin and tualang honey (F), glibenclamide (G), glibenclamide and tualang honey (H), metformin and glibenclamide (I) or metformin, glibenclamide and tualang honey (J). In spite of treatment, all these sections—Figure 2(D–J) —show necrosis of the islets of Langerhans, cellular degeneration and a reduced number of cells compared to Figure 2A and 2B. However, compared to Figure 2C, these sections—Figure 2(D–J) —show some improvements as characterized by less severe damage, partial restoration of cellular population and enlarged size of the islets of Langerhans.

In view of the fact that diabetes is a disorder of multiple defects, monotherapy with hypoglycemic agents which target a specific pathological condition may not be effective. This perhaps contributes to the increased morbidity and mortality associated with this disorder. Based on current literature, it is noteworthy that this is the first study which investigates and compares the effect of an antioxidant (honey) in combination with glibenclamide, metformin or glibenclamide + metformin on antioxidant enzymes and other markers of oxidative stress in kidneys of STZ-induced diabetic rats.

Lipid peroxidation, measured as malondialdehyde (MDA), reflects the impact of oxidative stress in cells and tissues. In diabetes, insulin deficiency enhances the activity of fatty acyl Coenzyme A oxidase, an enzyme that causes oxidation of fatty acids, leading to increased hydrogen peroxide formation [16]. Peroxides, including hydrogen peroxide, are known to exert detrimental effects on proteins, lipids, and polyunsaturated fatty acids (PUFAs) of cellular membranes. These deleterious effects can occur directly and/or indirectly through the formation of highly reactive hydroxyl radical or reaction with transition ions such as copper or iron to form toxic aldehydes [17]. Lipid peroxidation products are also vulnerable to radicals, thus further propagating free radical generation and contributing to oxidative damage [18]. They can also damage deoxyribonucleic acid (DNA) by forming cross links with it [19]. In this study, renal MDA concentrations in diabetic rats were significantly elevated. This is similar to what was previously reported [14]. The increased MDA levels suggest the occurrence of lipid oxidative damage which is implicated in the development of diabetic nephropathy [18]. Treatment with glibenclamide, metformin, honey, and their combinations significantly reduced the levels of MDA. These findings suggest that these agents exhibit anti-peroxidative effect. This may be due to their hypoglycemic effect as previously reported and also antioxidant properties in the case of tualang honey [7,14,20,21].

The measurement of total antioxidant status (TAS) indicates the total summation of the individual enzymatic and non-enzymatic antioxidants present in a sample [22]. Thus, TAS may be altered during oxidative stress. The reduced TAS in the untreated diabetic rats might imply that there was an imbalance between free radical formation and renal antioxidant protection. This might be a consequence of increased utilization of renal endogenous antioxidants in response to elevated levels of free radicals. Our data showed that none of the hypoglycemic agents elevated TAS. On the other hand, combination of metformin or glibenclamide with tualang honey significantly increased TAS. This might demonstrate that tualang honey offered additional antioxidant effect to these drugs.

Glutathione, an important intracellular free radical scavenger and co-substrate for many important enzymes, plays a prominent role in the degradation of hydrogen peroxide undergoing oxidation from its reduced form (GSH) to an oxidized state (GSSG) [23]. The ratio of GSH to GSSG often reflects cellular redox balance. A low level of GSH is reported in diabetes mellitus [13]. The reduced levels of GSH and GSH/GSSG ratio of the diabetic rats would indicate that the renal glutathione defense system was significantly compromised. The high levels of MDA in the diabetic rats might also contribute to the impaired glutathione defenses [13]. In view of the fact that GSH content, GSH/GSSG ratio, and TAS are all indicators for antioxidant capacity of a tissue to protect itself against oxidative stress and damage caused by free radicals, it could be inferred that the kidneys of diabetic rats are highly vulnerable to oxidative stress as reported in this study and others [24,25]. Our results showed that, unlike metformin or honey, glibenclamide did not produce any effect on GSH or GSH/GSSG ratio. However, in combination with tualang honey, glibenclamide increased GSH. Hence, this suggests the beneficial effect of tualang honey on glibenclamide.

Our results showed that superoxide dismutase (SOD) activity was increased while catalase (CAT) activity was reduced in the kidneys of STZ-induced diabetic rats. There are conflicting results with regard to SOD or CAT status in diabetes. Some studies reported reduced activities of these enzymes while others found increase [13]. Treatment with antioxidants such as α-lipoic acid, vitamins C and E was reported to reverse the activities of these enzymes while in other studies no changes were observed with antioxidant treatment [13]. Diabetes mellitus is often characterized by chronic hyperglycemia [2]. In the mitochondria, the excessive levels of glucose lead to an overdrive of the electron transport chain, resulting in the formation of excess superoxide anions [26]. Thus, the elevated blood glucose in the diabetic control rats might enhance production of superoxide radical. Oxidative stress ensues when SOD, which normally scavenges superoxide, becomes overwhelmed. The role of SOD is certainly important in the regulation of oxidative stress in diabetes mellitus. Therefore, increased SOD activity might be a response to increased generation of superoxide anions. Enhanced activity of antioxidant enzymes has been reported as an adaptive mechanism to protect cells against the toxicity of free radicals [27]. Since SOD converts superoxide anions to hydrogen peroxide (H_2_O_2_), enhanced SOD activity might lead to increased turnover of H_2_O_2_. Normally, H_2_O_2_ is further metabolized to H_2_O and O_2_ by CAT. CAT, being an endogenous antioxidant enzyme, will need to be replenished. However, in the event of increased generation of H_2_O_2_, CAT might be unable to scavenge sufficiently the high levels of H_2_O_2_ and thus become inhibited. Besides, CAT is reported to be highly susceptible to increased superoxide anions [28]. Therefore, all these might be responsible for the reduced activity of CAT observed in the diabetic kidney.

Glutathione peroxidase (GPx) plays an important role in the metabolism of hydrogen and lipid peroxides by using reduced glutathione (GSH) as a hydrogen donor resulting in the formation of oxidized glutathione (GSSG) [23]. On the other hand, glutathione reductase (GR) helps to regenerate glutathione by recycling GSSG back to GSH using NADPH as a co-factor [23]. In diabetes, glutathione peroxidase activity is reported to be increased in many tissues including kidney [13]. Treatment with antioxidants such as piperine restored GPx activity while boldine or quercetin did not [13]. In this study, GPx activity was significantly increased while GR activity was reduced insignificantly in diabetic kidneys. The increased GPx activity might be a consequence of increased H_2_O_2_ generated by enhanced SOD activity. This is often a compensatory mechanism to protect tissues against toxic effect of organic and inorganic peroxides including excess H_2_O_2_ [29]. The reduced activity of GR may indicate the inability of the diabetic kidneys to effectively regenerate GSH from GSSG. This impaired recycling of GSSG to GSH might contribute to low levels of GSH and GSH/GSSG ratio in the diabetic rats. Michiels and his colleagues have showed that the protection of cells against oxidative stress and damage is more effective when antioxidant enzymes act together [30]. Therefore, the occurrence of uncoordinated activities of these antioxidant enzymes in the diabetic kidneys might imply that the diabetic rats were not protected against oxidative stress. Even though GPx activity remained increased in all the treated rats, the beneficial effect of tualang honey in combination with metformin or glibenclamide on CAT activity might contribute in reducing excess H_2_O_2_ levels. This beneficial effect on these drugs might be responsible for the increased GSH, TAS and GR.

As previously reported, both the kidneys and pancreas of diabetic rats are subject to oxidative stress [14,15,31]. This present study suggests that metformin or glibenclamide might ameliorate oxidative stress in the kidneys of diabetic rats to a certain extent with regard to SOD and MDA. However, these drugs do not produce a similar antioxidant effect in the pancreas as we previously reported [32,33]. Although all the drugs showed a beneficial effect on the histology of the kidney and pancreas, our previous findings [32,33] as well as these current data seem to imply that these hypoglycemic agents when administered alone might not delay free radical-mediated damage in both pancreas and kidneys.

Until now, an intensive treatment of hyperglycemia is thought to be of immense benefits in both type 1 and type 2 diabetic patients [34,35]. However, recent findings have proved otherwise [36]. Ismail-Beigi and his colleagues carried out a study where they investigated the effect of intensively-treated hyperglycemia on the rate of microvascular complications in type 2 diabetic patients [36]. Besides hypoglycemia and weight gain, they discovered that mortality was higher in the intensive treatment group than in the standard group. Hence, intensive treatment was discontinued before the end of the study while patients were transitioned to the standard group. In light of these remarkable findings, it can be speculated that the higher mortality rate in the intensively-treated group may further corroborate the ineffectiveness, limitations and toxicities of these hypoglycemic drugs in the management of diabetes mellitus. Their findings also lend credence to the notion that the management of diabetes mellitus and its complications should not be restricted to hyperglycemia alone. Taken together, the relevance of our findings is that it is high time clinical studies involving a combination of metformin or glibenclamide and specific antioxidants were performed. The hypoglycemic effect of tualang honey as reported earlier [14] coupled with its potentially protective effects on the pancreas [15,32,33] and kidneys against oxidative damage would appear to make it a suitable candidate for such adjunctive use.

## 3. Materials and Methods

### 3.1. Animals and Chemicals

The study protocol was approved by the Animal Ethics Committee of Universiti Sains Malaysia. All animal procedures were performed in strict compliance with the Institutional Guidelines for the Care and Use of Animals for Scientific Purposes and in accordance with the Recommendations from Helsinki Declaration. Male Sprague-Dawley rats aged 12–14 weeks were used in this study. The rats were obtained from the Laboratory Animal Research Unit of Universiti Sains Malaysia, Health Campus, Kelantan, Malaysia. They were acclimatized to a well ventilated animal room at 25 ± 2 °C with 12-h light/12-h dark cycles for at least a week prior to the experiment. All animals were supplied with commercial pellet food and water *ad libitum*.

Streptozotocin, glibenclamide, thiobarbituric acid (TBA), dimethyl sulfoxide and glutathione reductase were obtained from Sigma–Aldrich (St. Louis, MO, U.S.). GSH:GSSG assay kit was purchased from Calbiochem (CA, U.S.). Superoxide dismutase and glutathione peroxidase assay kits were purchased from Cayman (MI, U.S.). Bio-Rad protein assay kit was purchased from Bio-Rad (U.S.). All other chemicals used were of analytical grade obtained from commercial sources.

Tualang honey (AgroMas^®^, Malaysia) was supplied by Federal Agricultural Marketing Authority (FAMA), Kedah, Malaysia. The honey has the following composition: total reducing sugar (67.5%) [fructose (29.6%), glucose (30.0%), maltose (7.9%); fructose/glucose ratio (0.99)], sucrose (0.6%) and water (20.0%). The dose (1.0 g/kg body weight) was chosen based on our previous study [13]. The honey was diluted with distilled water and prepared freshly each time it was administered. Glibenclamide and metformin were dissolved in dimethyl sulfoxide (DMSO) and distilled water, respectively, before administration.

### 3.2. Induction of Experimental Diabetes

Diabetes was induced in overnight fasted rats by a single intraperitoneal injection of STZ in a 0.1 M sodium citrate buffer (pH 4.5). The age-matched control rats received an equivalent amount of citrate buffer. Food and water intake were closely monitored daily after STZ administration. The development of hyperglycemia in rats was confirmed by measurement of fasting (16 h) blood glucose 48 h after STZ administration with the aid of a portable glucometer (Accu-Chek, Roche, Germany) using a drop of blood from the tail vein. The animals showing fasting blood glucose level ≥14.0 mmol/L with other symptoms of diabetes mellitus such as polyphagia, polydipsia, polyuria, and weight loss were considered diabetic and included in the study.

### 3.3. Treatment

The animals were randomly divided into ten groups. Each group comprised five to seven rats. Distilled water, tualang honey, glibenclamide, metformin or their combinations were administered once daily by oral gavage for 4 weeks as follows:

Group 1: Non-diabetic + Distilled water (0.5 mL)
Group 2: Non-diabetic + Tualang honey (1.0 g/kg body weight)
Group 3: Diabetic + Distilled water (0.5 mL)
Group 4: Diabetic + Tualang honey (1.0 g/kg body weight)
Group 5: Diabetic + Metformin (100 mg/kg body weight)
Group 6: Diabetic + Metformin (100 mg/kg body weight) + Tualang honey (1.0 g/kg body weight)
Group 7: Diabetic + Glibenclamide (0.6 mg/kg body weight)
Group 8: Diabetic + Glibenclamide (0.6 mg/kg body weight) + Tualang honey (1.0 g/kg body weight)
Group 9: Diabetic + Glibenclamide (0.6 mg/kg body weight) + Metformin (100 mg/kg body weight)
Group 10: Diabetic + Glibenclamide (0.6 mg/kg body weight) + Metformin (100 mg/kg body weight) + Tualang honey (1.0 g/kg body weight)

Fasting blood glucose and body weight were measured weekly. After 4 weeks of treatment, the animals were fasted overnight and sacrificed by decapitation. The kidneys were excised and processed.

### 3.4. Processing of Tissues

The left kidney was rapidly excised, washed in ice-cold normal saline, blotted, frozen in liquid nitrogen, and stored at −80 °C until use. Similarly, the right kidney and pancreas were excised, fixed in 10% formaldehyde, and dehydrated in ascending grades of ethanol, cleaned in xylene and embedded in paraffin. Sections (5 mm thick) were cut and stained with hematoxylin and eosin (H&E) before the slides were subjected to photomicroscopic observation. Digital images were obtained from a high-resolution digital camera system (Penguin 150CL, Pixera, Los Gatos, CA, U.S.) linked to a microscope (BX41, Olympus, Tokyo, Japan) and desktop computer (Pentium 4, 2.0 GHz). Images of glomeruli at 100× magnification were digitized.

Frozen kidneys were thawed and homogenized to make 10% homogenate (w/v) in ice-cold Tris-HCl (0.1 M, pH 7.4) using an ice-chilled glass homogenizing vessel in a homogenizer fitted with Teflon pestle (Glas-Col, U.S.) at 900 rpm. The homogenates were centrifuged at 1000 × g for 10 min at 4 °C in a refrigerated centrifuge to remove the nuclear debris. The resulting supernatants were used for the assay of total protein, levels of MDA, activities of enzymatic and non-enzymatic antioxidants.

### 3.5. Assay of Reduced and Oxidized Glutathione

Reduced and oxidized glutathione as well as reduced: oxidized glutathione (GSH:GSSG) ratio were estimated using Calbiochem GSH:GSSG ratio kit according to the manufacturer’s instructions. Briefly, the kidney homogenates were deproteinized in 5% metaphosphoric acid, centrifuged and the glutathione contents of the supernatants were measured by the rate of colorimetric change of 5,5′-dithiobis(nitrobenzoic acid) at 412 nm in the presence of glutathione reductase and NADPH.

### 3.6. Superoxide Dismutase (SOD) Assay

Superoxide dismutase (SOD) activity was measured using Cayman (MI, U.S.) assay kit according to the manufacturer’s instructions. This assay kit utilizes a tetrazolium salt for the detection of superoxide radicals generated by xanthine oxidase and hypoxanthine. One unit of SOD is defined as the amount of enzyme needed to exhibit 50% dismutation of superoxide radical. The SOD assay measures all the three types of SOD (Cu/Zn, Mn, and FeSOD).

### 3.7. Glutathione Peroxidase (GPx) Assay

Glutathione peroxidase (GPx) activity was measured using Cayman (MI, U.S.) assay kit according to manufacturer’s instructions. This kit measures GPx activity indirectly by a coupled reaction with glutathione reductase (GR). Oxidized glutathione (GSSG), produced upon reduction of hydroperoxide by GPx, is recycled to its reduced state by GR and NADPH. The oxidation of NADPH is accompanied by a decrease in absorbance at 340 nm. One unit of GPx is defined as the amount of enzyme that catalyzes the oxidation of 1 nmol of NADPH per minute at 25 °C.

### 3.8. Catalase (CAT) Assay

CAT activity was measured according to the method of Goth [37]. Briefly, this assay involves the incubation of 0.5ml of hydrogen peroxide and 0.1 mL of kidney homogenate in a sample test tube. After incubation at 37 °C for 60 sec, the enzymatic reaction was stopped by addition of 0.5 mL of ammonium molybdate solution. The yellow complex of ammonium molybdate and hydrogen peroxide was then measured spectrophotometrically at 405 nm. One unit of CAT was defined as the amount of enzyme that catalyzes the decomposition of 1 μmol of hydrogen peroxide per minute.

### 3.9. Glutathione Reductase (GR) Assay

Glutathione reductase (GR) activity was assayed according to the method of Goldberg and Spooner [38]. Briefly, 1 mL of 2.728 mM GSSG solution and 40 μL of kidney homogenate were incubated for 5 min at 37 °C. After incubation, the reaction was initiated by addition of 200 μL of 1.054 mM NADPH solution. The decrease in absorbance was measured at 340 nm using a spectrophotometer and recorded every 30 sec over a period of 5 min. GR activity was expressed as unit per mg protein based on molar extinction coefficient of 6.22 × 10^3^ L mol^−1^ cm^−1^. One unit of GR was defined as the amount of enzyme that catalyzes the oxidation of 1 nmol of NADPH per minute.

### 3.10. Glutathione-S-Transferase (GST) Assay

Glutathione-S-transferase (GST) activity was assayed according to the method of Habig *et al.* [39]. Briefly, 2 mL of 0.3 M potassium phosphate buffer (pH 6.35), 75 μL of 30 mM CDNB solution, 725 μL of distilled water and 0.1 mL of kidney homogenate were pipetted into a test tube. The test tube was mixed using a vortex and incubated at 37 °C for 10 min. After incubation, the reaction was initiated by addition of 100 μL of 30 mM reduced glutathione solution. The decrease in absorbance was measured spectrophotometrically at 340 nm and recorded every 30 sec for 4 min. GST activity was calculated as unit per mg protein based on a molar extinction coefficient of 9.6 × 10^3^ L mol^−1^ cm^−1^. One unit of GST was defined as the amount of enzyme that catalyzes the conjugation of 1 nmol of GSH-CDNB per minute.

### 3.11. Lipid Peroxidation Assay

The extent of lipid peroxidation was determined as the concentration of malondialdehyde (MDA) accordin*g* to the method of Ohkawa *et al.* [40]. Briefly, 100 μL of kidney homogenates or MDA standards were pipetted into test tubes containing 1.5 mL of 20% (w/v) glacial acetic acid (pH 3.5), 200 μL of 8.1% (w/v) sodium dodecyl sulphate (SDS), 1.5 mL of 0.8% (w/v) thiobarbituric acid (TBA) and 700 μL of distilled water. The test tubes were incubated at 95 °C for 60 min with a marble on top of each test tube. After incubation, the test tubes were cooled and then centrifuged at 3000 × g for 10 min. The amount of malondialdehyde (MDA) formed was measured spectrophotometrically at 532 nm. 1,1,3,3-Tetraethoxypropane (TEP), a form of MDA, was used as standard in this assay. TBARS concentration was expressed as nmol of malondialdehyde (MDA) per mg protein.

### 3.12. Total Antioxidant Status (TAS) Assay

TAS was measured according to the method of Koracevic *et al.* [41]. Briefly, 10 μL of kidney homogenate was pipetted in a test tube containing 0.49 mL of 100 mM sodium phosphate buffer. This was followed by the addition of 0.5 mL of 10 mM sodium benzoate solution, 0.2 mL of Fe-EDTA mixture prepared from 2 mM EDTA solution and 2 mM Fe(NH_4_)_2_(SO_4_)_2_ solution and 0.2 mL of 10 mM H_2_O_2_ solution. Each sample had its own control (blank) in which 1 mL of 20% acetic acid was added followed by the addition 0.2 mL of Fe-EDTA mixture and 0.2 mL of 10 mM H_2_O_2_. Negative control was also prepared. After the reagents were added, the test tubes were vortexed and incubated at 37 °C for 60 min. This was followed by the addition of 1 mL of 20% acetic acid (sample test tubes only) and TBA. The reaction tubes were incubated at 100 °C for 10 min. After cooling to room temperature, the absorbance was measured spectrophotometrically at 532 nm against distilled water. TAS in the kidney homogenates was calculated using uric acid as standard.

### 3.13. Protein Assay

Protein concentration was estimated using a Bio-Rad protein assay kit based on the method of Bradford [42]. The assay is a dye-binding assay in which a differential color change of a dye, with maximum absorbance at 595 nm, occurs in response to various concentrations of protein.

### 3.14. Statistical Analysis

Data were analyzed using SPSS 18.0.1. The data are expressed as median (interquartile range). Groups were compared by Kruskal-Wallis *H* test. Differences between two groups were identified by Mann-Whitney *U* test followed by Bonferonni’s correction.

## 4. Conclusions

These data suggest that the hypoglycemic drugs, metformin or glibenclamide, when administered alone might not efficiently arrest oxidative stress mediated damage in the kidneys of diabetic rats. Our findings demonstrate the beneficial role of tualang honey as an adjunct to metformin or glibenclamide in ameliorating oxidative stress in the kidneys of streptozotocin-diabetic rats. Further studies are needed in human subjects to determine if these results can be appropriately extrapolated to human diabetes. This may result in better and more efficient management of diabetes mellitus and its related complications.

## Figures and Tables

**Table 1:** Effects of honey, metformin, glibenclamide or their combinations on fasting blood glucose and antioxidant enzymes in the kidneys of control and streptozotocin-induced diabetic rats.

Group	FBG (mmol/L)	SOD (U/mg Protein)	CAT (U/mg Protein)	GPx (U/mg Protein)	GR (U/mg Protein)	GST (U/mg Protein)
Non-diabetic + Distilled water	4.0 (0.9)	1.03 (1.13)	444.2 (89.2)	322.0 (124.9)	208.0 (74.8)	182.5 (50.8)
Non-diabetic + Honey	3.7 (0.7)	0.96 (0.69)	463.2 (131.8)	326.1 (105.7)	195.9 (38.1)	158.4 (72.1)
Diabetic + Distilled water	17.9 (2.6) **	2.06 (0.23) *	239.4 (61.6) **	468.7 (47.9) **	175.6 (29.4)	174.0 (30.1)
Diabetic + Honey	8.8 (5.8) †	1.04 (0.45) †	347.6 (65.0) †	438.7 (93.8)	223.8 (29.3) †	185.2 (56.6)
Diabetic + Metformin	8.4 (6.1) *,†	1.15 (0.40) †	286.5 (76.0)	499.9 (185.5)	213.1 (61.3)	177.9 (73.6)
Diabetic + Metformin + Honey	8.5 (6.8) *,†	1.55 (0.64) †	341.2 (89.7) †	550.5 (80.8)	220.6 (15.1) †	172.7 (31.6)
Diabetic + Glibenclamide	13.5 (10.4) *,†	1.02 (0.76) †	297.7 (149.2)	494.5 (115.6)	223.6 (59.3)	172.0 (64.6)
Diabetic + Glibenclamide + Honey	12.3 (6.7) *,†	1.08 (0.54) †	352.7 (54.9) †	472.0 (47.1)	239.3 (31.3) †	189.2 (41.1)
Diabetic + Metformin + Glibenclamide	7.9 (6.5) †	0.96 (0.80)	299.3 (49.6)†	527.2 (91.2)	214.1 (49.4)	173.4 (37.9)
Diabetic + Metformin + Glibenclamide + Honey	6.8 (8.4)	1.45 (1.07)	330.6 (80.1)	448.8 (225.3)	200.0 (90.9)	164.7 (96.3)

Data are expressed as median (interquartile range). Each group consisted of 5–7 rats. Groups were compared by Kruskal-Wallis *H* test. Differences between two groups were identified by Mann-Whitney *U* test followed by Bonferonni’s correction. Values are statistically significant at

* *p* < 0.05,

** *p* < 0.01 compared to non-diabetic + distilled water;

† *p* < 0.05 compared to diabetic + distilled water. FBG, fasting blood glucose; SOD, superoxide dismutase; CAT, catalase; GPx, glutathione peroxidase; GR, glutathione reductase; GST, glutathione-S-transferase. One unit of SOD = the amount of enzyme required to exhibit 50% dismutation of superoxide radical. One unit of CAT = the amount of enzyme that catalyzes the decomposition of 1 μmol of H_2_O_2_ per minute. One unit of GPx/GR = the amount of enzyme that catalyzes the oxidation of 1 nmol of NADPH per minute. One unit of GST = the amount of enzyme that catalyzes the conjugation of 1 nmol of GSH-CDNB per minute.

**Table 2:** Effects of honey, metformin, glibenclamide or their combinations on markers of oxidative stress in the kidneys of control and streptozotocin-induced diabetic rats.

Group	TAS (nmol/mg Protein)	MDA (nmol/mg Protein)	GSH (nmol/mg Protein)	GSSG (nmol/mg Protein)	GSH/GSSG
Normal + Distilled water	1.03 (1.19)	1.43 (0.32)	0.140 (0.021)	0.055 (0.015)	2.65 (0.29)
Normal + Honey	0.97 (1.60)	1.29 (0.62)	0.132 (0.095)	0.049 (0.025)	2.56 (1.05)
Diabetic + Distilled water	0.52 (0.21) *	2.10 (0.91) **	0.044 (0.012) **	0.047 (0.006)	1.03 (0.95) *
Diabetic + Honey	0.70 (0.32)	1.02 (0.21) †	0.121 (0.043) †	0.045 (0.017)	2.49 (1.23)
Diabetic + Metformin	0.70 (0.15)	1.08 (0.72) †	0.140 (0.053) †	0.052 (0.015)	2.66 (1.51)
Diabetic + Metformin + Honey	0.85 (0.09) †	1.18 (0.19) †	0.117 (0.053) †	0.049 (0.028)	1.90 (0.52)
Diabetic + Glibenclamide	0.80 (0.30)	1.43 (0.59) †	0.078 (0.082)	0.043 (0.027)	2.10 (1.54)
Diabetic + Glibenclamide + Honey	0.83 (0.27) †	1.31 (0.86) †	0.116 (0.042) †	0.052 (0.052)	2.01 (1.10)
Diabetic + Metformin + Glibenclamide	0.73 (0.17)	1.39 (0.26) †	0.100 (0.066)	0.037 (0.012)	2.43 (1.47)
Diabetic + Metformin + Glibenclamide + Honey	0.78 (0.19)	1.36 (0.78) †	0.113 (0.121)	0.049 (0.019)	1.61 (2.25)

Data are expressed as median (interquartile range). Each group consisted of 5–7 rats. Groups were compared by Kruskal-Wallis *H* test. Differences between two groups were identified by Mann-Whitney *U* test followed by Bonferonni’s correction. Values are statistically significant at

* *p* < 0.05,

** *p* < 0.01 compared to non-diabetic + distilled water;

† *p* < 0.05 compared to diabetic + distilled water. TAS, total antioxidant status; MDA, malondialdehyde; GSH, reduced glutathione; GSSG, oxidized glutathione.
